# First detection of hepatitis E virus in dromedary camels from Iran

**DOI:** 10.1002/vms3.1174

**Published:** 2023-06-08

**Authors:** Ali Sarani, Atefeh Ravanbakhsh, Hossein Kamaladini

**Affiliations:** ^1^ Clinical Sciences Department, Faculty of Veterinary Medicine University of Zabol Zabol Iran; ^2^ General Veterinary Medicine Graduate, Faculty of Veterinary Medicine University of Zabol Zabol Iran; ^3^ Biology Department, Faculty of Science University of Zabol Zabol Iran

**Keywords:** dromedary camel, hepatitis E virus, Iran, RT‐PCR, zoonosis

## Abstract

**Background:**

Hepatitis E virus (HEV) genotype 7 is a zoonotic disease detected in dromedary camels.

**Hypothesis/objectives:**

The consumption of camel meat and dairy products, the abundance of dromedary camels in Southeast Iran and the import of camels from neighbouring countries to Iran made the researchers investigate the infection rate of camels by the virus.

**Animals:**

A total of 53 healthy camels in Southeast Iran (Sistan and Baluchistan Province) tested for HEV RNA.

**Method:**

A total of 17 blood samples and 36 liver samples were taken from 53 healthy dromedary camels (aged between 2 and 10 years) from various southeastern regions of Iran. The samples were tested for HEV using RT‐PCR.

**Results:**

Overall, 56.6% of the studied samples (*n* = 30) tested positive for HEV RNA.

**Conclusions and clinical importance:**

The present study was the first of its kind in Iran and revealed the presence of HEV in the Iranian dromedary camel population, which might play the role of a zoonosis reservoir for its transmission to humans. This discovery raises concerns about food‐borne illnesses that can be transmitted from animals to humans. However, further research is needed to identify the specific genotype of the HEV in Iranian dromedary camel infections and to determine the risk of spread to other animals and humans.

## Graphical abstract

The present study was the first of its kind in Iran and revealed the presence of Hepatitis E virus (HEV) from dromedary camels in Southeast Iran using reverse transcription polymerase chain reaction, which might play the role of a zoonosis reservoir for its transmission to humans.

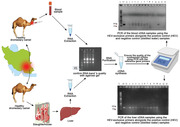

## 1 INTRODUCTION

Hepatitis E virus (HEV) is among the leading causes of acute hepatitis worldwide (Rasche et al., 2016; Woo et al., 2016). According to the World Health Organization statistics, over 20 million new cases with these diseases are observed annually worldwide (Taherkhani & Farshadpour, 2016). This pathogen is mainly transmitted via contaminated water and food, faecal–oral, blood, zoonosis, vertical transmission and organ transplant. Wild boars and domestic pigs are the leading animal reservoirs transmitting the virus (Khuroo et al., 2016). This disease is asymptomatic in most individuals and often creates self‐limiting jaundice after the onset of the symptoms (Bari et al., 2021; Taherkhani & Farshadpour, 2016; Woo et al., 2014). The disease might be manifested more severely with acute liver failure syndrome in pregnant women, infants and patients suffering from liver damage. Moreover, patients suffering from immunodeficiency might develop chronic hepatitis after infection with HEV (Bari et al., 2021; Meng, 2010; Woo et al., 2016, 2014). The mortality rate caused by the HEV infection is 1%–2% in the general population; however, it increases up to 10%–25% in pregnant women and above 70% in patients suffering from liver damages (Taherkhani & Farshadpour, 2016). The HEV strains remain silent and subclinical in most animal species and represent no clinical symptoms; however, the infected animals might indicate microscopic hepatitis evidence (Meng, 2010).

According to the genomic sequence analysis, the HEV strains from humans and other mammals are categorized into eight genotypes. The strains of HEV genotypes 1 and 2 only infect humans, are endemic in developing countries and are primarily transmitted via the faecal–oral route. In contrast, strains from genotypes 3 and 4 have a broader host range and geographical spread and circulate among humans, wild boars, pigs, deer, mongoose, macaques, sheep, cows, yaks and rabbits, which are the sporadic agents of human hepatitis E in underdeveloped and industrial countries (Aggarwal, 2011). These strains induce silent infections in various mammals and are sometimes transmitted to humans. HEV‐5 and HEV‐6 are the new genotypes detected in Japanese wild boars. The HEV‐7 and HEV‐8 genotypes have also recently been detected in dromedary and Bactrian camels, respectively (Sridhar et al., 2017). HEV was first identified in dromedary camels in the Middle East in 2014 and was classified in genus Orthohepevirus A under dromedary Camel HEV (DcHEV) (Woo et al., 2014). The transmission of HEV‐7 to nonhuman primates and to a person in the UAE who consumed infected camel products confirmed its zoonotic nature (Lee et al., 2016; Li et al., 2016). Furthermore, a long‐term epidemiological study revealed that dromedary camels have long suffered from DcHEV and that the virus is geographically extensive and diverse (Rasche et al., 2016). Hepatitis E is endemic in many Middle Eastern countries such as Iran; however, the presence of HEV RNA has not been addressed in previous studies. Accordingly, the distribution pattern of the HEV genotypes is still unknown in Iran (Khuroo et al., 2016; Taherkhani & Farshadpour, 2016), and the zoonotic role of animals has yet to be investigated in this regard. The increase in camel meat and dairy consumption, the abundance of dromedary camels in Southeast Iran and the import of camels from Pakistan and Afghanistan to these regions highlight the need for studies examining the infection rate of the virus among dromedary camels through reverse transcription polymerase chain reaction (RT‐PCR). The present study was the first of its kind in Iran.

## 2 MATERIALS AND METHODS

Different stages of the study were approved on 26/12/2018 by the Ethics Committee for Working with Animals (Code: IRUOZ.ECRA.2018.003). The southeastern region of Iran (Sistan and Baluchistan Province) was selected for the study as half of dromedary camels in Iran and camels imported from Pakistan and Afghanistan are in this region. The samples were taken randomly from camels in Zabol, Saravan and Mirjaveh Counties, Sistan and Baluchistan province with the largest camel population. Sampling was carried out over multiple periods during January–June 2019. The blood samples were taken from the jugular vein of apparently healthy dromedary camels after clinical examinations and with the consideration of the ethical principles, and liver samples were taken from apparently healthy camels after slaughter. Each sample's information, including age, gender and geographical location, was collected and recorded using specific questionnaires.

**FIGURE 1:**
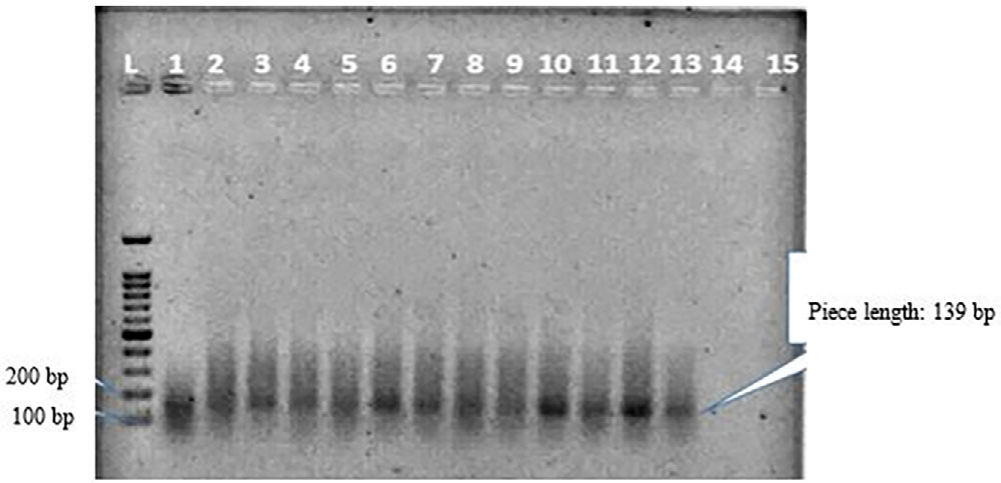
PCR results of cDNA samples using reference genes to ensure their quality. L: ladder 50 bp, No. 1–15: PCR results using reference gene. The reference gene fragment had a length of 139 bp.

**FIGURE 2:**
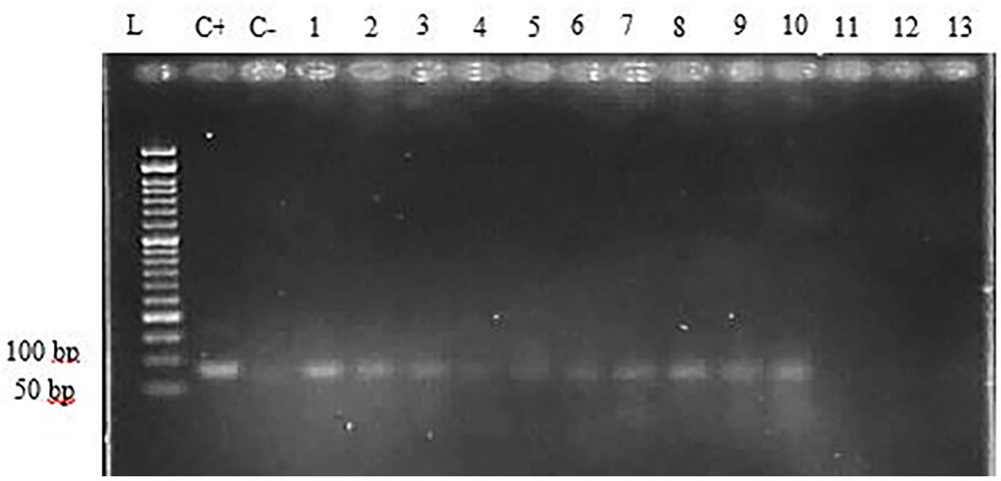
PCR results of camel liver cDNA samples using specific HEV positive control primers. L: ladder 50 bp, C+: HEV positive control sample, C−: HEV negative control sample, nos. 1–3: positive liver samples, no. 4: negative liver sample, nos. 5–10 positive liver samples, nos. 11–13: negative liver sample. Length of HEV positive control fragment: 80 bp.

**FIGURE 3:**
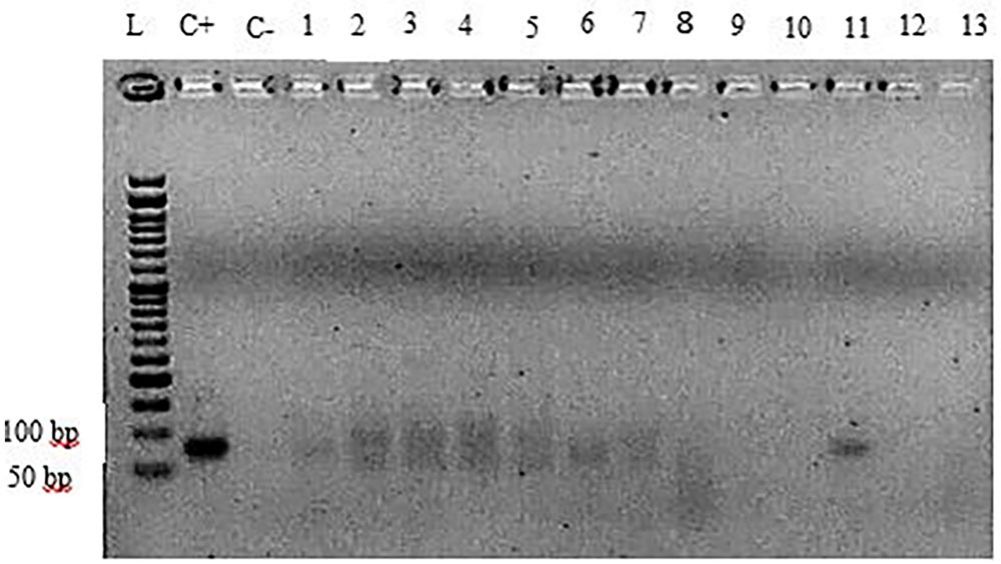
PCR results of camel blood cDNA samples using specific HEV positive control primers. L: ladder 50 bp, C+: HEV positive control, C−: negative control sample, nos. 1, 2, 3, 4, 6, 7 and 11: positive blood sample. Length of HEV positive control fragment: 80 bp.

Seventeen blood samples and 36 liver samples were obtained from apparently healthy dromedary camels (aged between 2 and 10 years) in Southeast Iran. About 58.5% of the camels were male, and 41.5% of them were female. The DENAzist column RNA isolation kit (DENAzist Asia Co., Mashhad, Iran) was used according to the manufacturer's instructions to extract RNA from the liver samples, and the DENAzist whole blood RNA isolation kit was used to extract RNA from the blood samples. Following the RNA extraction, 5 μL of the extracted RNA along with 1 μL of loading buffer‐dye 6X were loaded onto 1.5% agarose gel to ensure the quality and non‐degradation of RNA bands. A NanoDrop device was used for the quantitative study of the extracted RNA. After confirming the RNA extracted from camel liver and blood, the RNA purification was carried out using the DNase (ZandBiotech, Co., Iran) commercial kit according to a kit protocol to eliminate possible DNA contaminations. The purified RNAs were then used for cDNA synthesis using the Pars Toos commercial kit. All stages, from RNA extraction to cDNA synthesis, were carried out immediately after sampling on the same day. The extracted samples were stored in freezers at −80°C for the subsequent examinations. The Actin Beta‐like2 gene (ACTBL2) was used as a reference gene to ensure high‐quality cDNA synthesis. The gene was found to be 100% present in dromedary camels after blast analysis. After alignment and thermodynamic evaluation of the primers, the forward and reverse gene reference primers 5′‐TATTGGCAACGAGCGGTTCC‐3′ and 5′‐GGCATAGAGGTCTTTACGGATGTC‐3′, respectively, were selected. An ordinary PCR was performed with the reference gene primers and synthesized cDNAs at 54°C to verify cDNA quality. After loading the result on agarose gel, the presence of bands in the range of the length of reference gene primers’ fragment (139 bp) indicated the quality of the synthesized cDNAs (Figure 1). Finally, the PCR of the cDNA samples was carried out using the HEV‐exclusive primers (5′‐GGTGGTTTCTGGGGTGAC‐3′ forward and 5′‐AGGGGTTGGTTGGATGAA‐3′ reverse) alongside the positive control (HEV) and negative control (distilled water) samples using a 95°C thermocycler program for 5 min in the first denaturation stage for 35 cycles at 95°C for 1 min, at 60°C for 1 min, at 72°C for 1 min and at 72°C for 7 min as the final stage. The positive control HEV sample was obtained from the Department of Microbiology, which was, in fact, a synthesized HEV sample with primers explicitly used for HEV (Hajiahmadi et al., 2017). The PCR products were loaded on a 2.5% agarose, run at 70 V for 70 min and imaged using a gel documentation system. PCR was repeated, and the results were recorded to confirm the positive samples (Figures 2 and 3). The data were then imported to SPSS software version 28 and analysed using the Fisher exact test.

## 3 RESULTS

Out of the 53 liver and blood samples obtained from apparently healthy camels in Southeast Iran, 56.6% (*n* = 30) tested positive for HEV RNA using the RT‐PCR method. Statistical data analysis using the Fisher exact test indicated a significant relationship between the camels’ hepatitis E infection and their geographical location (*p* < 0.001) so that the infection rate was higher in camels from Mirjaveh County than in those from other counties. The results also indicated the high frequency of infection in camels imported from Pakistan and Afghanistan. The camels’ infection with hepatitis E also revealed a significant relationship with their age (*p* < 0.001) so that the highest infection rate was observed in camels younger than 2 years old. The infection rate in the liver tissue was 65.71% (23/35) as opposed to the 38.89% (7/18) infection in the blood; however, the infection rate showed no significant relationship with the type of sample (*p* = 0.058). The frequency of HEV was revealed to be 67.74% (21/31) in male dromedary camels as opposed to 40.90% (9/22) in female dromedary camels; this difference was statistically significant (*p* = 0.048) (Table 1).

**TABLE 1:** Hepatitis E frequency in camels, divided by the factors of sample type, geographical location, gender, age and their impacts.

Variable	Category	Positive/total	Prevalence	*p Value*	Odds Ratio	95% Confidence Interval
Geographical location	Domestic	1/18	% 5.56	<0.001	54.13	[6.30–464.85]
	Imported from Pakistan (Saravan)	17/23	% 73.91			
	Imported from Afghanistan (Mirjave)	12/12	% 100.00			
Tissue	Blood	7/18	% 38.89	=0.058	3.01	[0.93–9.77]
	Liver	23/35	% 65.71			
Gender	Female	9/22	% 40.90	=0.048	3.03	[0.97–9.44]
	Male	21/31	% 67.74			
Age group (years)	Under 2 years	12/12	% 100.00	<0.001	1.06	[0.744–14.39]
	2–4 years	1/12	% 8.34			
	4–6 years	4/10	% 40.00			
	6–8 years	4/7	% 57.14			
	8–10 years	1/2	% 50.00			
	Over 10 years	8/10	% 80.00			

## 4 DISCUSSION AND CONCLUSION

Hepatitis E, known as a zoonotic disease, can be transmitted among humans, pigs, boars, deer, camels and cattle. The consumption of undercooked or raw animal products is the essential zoonosis source of this disease. The HEV infection is quickly spreading among various animals, to the point that it has been detected in mongoose, macaques, sheep, yaks, birds, bats, rabbits, rodents, minks, ferrets, fish, foxes and horses (Bari et al., 2021; Woo et al., 2014).

The present study collected blood and liver samples of apparently healthy dromedary camels in Southeast Iran to test their infection rate with HEV. The samples were tested using RT‐PCR. As the liver is the primary target tissue for the virus, a more significant number of liver samples were examined in the present study, and the molecular technique was selected for testing due to the high sensitivity of the PCR technique. As it is a diverse, extremely sensitive and fast method to investigate the expression of genes and differentiate small copies of RNA molecules, although an additional anti‐HEV test must be performed to confirm the infection (Al‐Sadeq et al., 2018).

Of the 53 liver and blood samples obtained from apparently healthy camels in Southeast Iran, 56.6% (*n* = 30) tested positive for HEV RNA. Previous reports indicated that the DcHEV prevalence was 1.5% (3 out of 203 stool samples) in the Middle East using the RT‐PCR method in 2014 (Woo et al., 2014) but 40% in Somalia, 15% in Sudan, 62.9% in Egypt, 31.4% in Kenya, 37.1% in the UAE and 60% in Pakistan using the enzyme‐linked immunosorbent assay (ELISA) method. Among them, a total of 0.6% of the dromedary camel serum samples (12 cases out of 2171 serum samples) and 1.9% of the dromedary camel stool samples (5 samples out of 267 stool samples) tested positive for HEV RNA using the RT‐PCR method (Rasche et al., 2016). Moreover, a study in Ethiopia in 2017 concluded that 22.4% of dromedary camel serum samples (55 cases out of 246 serum samples) tested positive for anti‐DcHEV IgG using the ELISA method (Li et al., 2017). Studies in 2019 reported that 8.33% of dromedary camels in the Sub‐Sahelian region (three West African countries, including Burkina Faso, Mali and Niger) tested positive for anti‐DcHEV IgG using the ELISA kit (Ouoba et al., 2019). Moreover, 68.6% of 86 serum samples from dromedary camels in Israel tested positive for anti‐HEV IgG using the ELISA kit. However, no serum sample tested positive for HEV RNA (Bassal et al., 2019). A study in 2020 revealed that the prevalence of DcHEV‐Abs in dromedary camel serum samples collected from Saudi Arabia was 23.1% (El‐Kafrawy et al., 2020). Another study in 2021 found that 2.2% of stool samples taken from dromedary camels in Ethiopia (one case out of 45 samples) tested positive for HEV RNA using the nested reverse transcription polymerase chain reaction. method (Bari et al., 2021). Moreover, in a study reported in 2022, DcHEV RNA was detected in 1.77% of dromedary camel blood serum samples (21/1189) in Saudi Arabia by RT‐PCR method (El‐Kafrawy et al., 2022).

Statistical analysis of our data revealed a significant relationship between the camels’ infection with HEV and their geographical location (*p* < 0.001). The tests indicated a higher frequency of infection in camels from border areas and camels imported from Pakistan and Afghanistan. Rasche et al. (2016) conducted a study on 2438 serum and stool samples from dromedary camels in six different countries (the UAE, Somalia, Egypt, Sudan, Kenya and Pakistan) using the ELISA method. They indicated a 60% infection in dromedary camels from Pakistan (Rasche et al., 2016). This finding is consistent with the high infection rate of camels imported from Pakistan in our study.

Our study revealed a possible correlation (*p* = 0.048) between HEV infection in male and female camels, with a higher number of infected males. El‐Kafrawy et al. (2020) reported a significant relationship between gender and the HEV prevalence in camels based on serum samples using the ELISA method (*p* = 0.015); hence, male camels were more likely to be infected than female camels (31.6% vs. 13.4%) (El‐Kafrawy et al., 2020). Similarly, a study conducted in Saudi Arabia found that all infected camels tested with RT‐PCR were male (El‐Kafrawy et al., 2022). Although our findings support the influence of gender on HEV infection in dromedary camels, some studies such as Li et al. (2017) and Ouoba et al. (2019) have not found any association between gender and HEV infection (Li et al., 2017; Ouoba et al., 2019). Therefore, further studies on risk factors are required in this regard.

Our study found a significant relationship between the distribution of camels’ hepatitis E infection and their age group so that the infection rate was significantly higher in camels under 2 years of age compared to other ages (*p* < 0.001). According to Corman et al., 2020, the natural process of the Genotype HEV‐7 infection in camels is similar to that of HEV‐3 in pigs in their first 6 months of life, which is associated with a decline in maternal antibody levels. This is consistent with the present study regarding the parameter of age. The study of El‐Kafrawy et al. in 2022 also reported the highest prevalence of HEV infection in young, single‐humped camels under 1 year of age (1/8, 12.5%), followed by the age group of 1–3 years (9/229, 3.9%), which is consistent with our study findings. Moreover, a recent study in the UAE showed that all camels were infected in the first 6 months of life and cleared the virus after an average of 2 months (Corman et al., 2020). As the natural course of HEV‐7 genotype infection in camels usually occurs in the first 6 months of life, the viremia period lasts on average 8 weeks (Corman et al., 2020), and camels are usually slaughtered at higher age (2 years old even in industrial farming) (Kadim et al., 2013), their meat products may have a less risky than pigs in terms of HEV transmission (Corman et al., 2020).

The present study was the first of its kind in Iran and revealed the presence of HEV in the Iranian dromedary camel population, which might play the role of a zoonosis reservoir for its transmission to humans. According to the religious consideration in Iran, boars and pigs are not bred and consumed in Iran and thus do not count as the main hepatitis E reservoirs in the country; as such, the role of other animals in the transmission of this infection must be investigated. More specifically, the consumption of camel milk and meat in the country and the reports of HEV transmission to humans via the consumption of camel products in the UAE indicate that the role of DcHEV in the human population of Iran must be specified. As HEV has been detected in the stool samples of camels (Bari et al., 2021; Rasche et al., 2016; Woo et al., 2014), camels can play a role in transmitting this virus to other animals and cause environmental problems. Accordingly, other wild or domestic animals in close contact with dromedary camels must be investigated to evaluate the HEV‐7 host range (Ouoba et al., 2019). The limitation of the present study is that the detection of anti‐DcHEV antibody from the collected samples was not carried out simultaneously to present the actual HEV infection rate, which must be considered in future studies. In this regard, the study of the HEV prevalence in the Iranian camel population and clarification of the disease load, the identification of the HEV genotype involved in dromedary camel infection and the evaluation of its zoonosis potential and transmission to other animals require further investigation. Moreover, further investigation should suggest gene sequencing and sequence analysis for detailed molecular analysis. Moreover, serological and molecular studies of the virus are recommended in other animal species and their various samples.

## AUTHOR CONTRIBUTIONS

*Conceptualization; methodology; project administration; supervision; writing – review and editing*: Ali Sarani. *Data curation; formal analysis; investigation; resources; validation; writing – original draft preparation*: Atefeh Ravanbakhsh. *Methodology; project administration; supervision; writing – review and editing*: Hossein Kamaladini.

## CONFLICT OF INTEREST STATEMENT

The authors declare no conflict of interest.

## FUNDING INFORMATION

This research did not receive any specific grant from agencies in the public, commercial or non‐profit sector.

## ETHICS STATEMENT

Different stages of the study were approved on 26/12/2018 by the Ethics Committee for Working with Animals (Code: IRUOZ.ECRA.2018.003).

### TRANSPARENT PEER REVIEW

The peer review history for this article is available at https://publons.com/publon/10.1002/vms3.1174.

## Data Availability

All the data is available upon requesting from the corresponding author.
